# *Vital Signs:* HIV Transmission Along the Continuum of Care — United States, 2016

**DOI:** 10.15585/mmwr.mm6811e1

**Published:** 2019-03-22

**Authors:** Zihao Li, David W. Purcell, Stephanie L. Sansom, Demorah Hayes, H. Irene Hall

**Affiliations:** 1 Division of HIV/AIDS Prevention, National Center for HIV/AIDS, Viral Hepatitis, STD, and TB Prevention, CDC.

## Abstract

**Background:**

In 2016, an estimated 1.1 million persons had human immunodeficiency virus (HIV) infection in the United States; 38,700 were new infections. Knowledge of HIV infection status, behavior change, and antiretroviral therapy (ART) all prevent HIV transmission. Persons who achieve and maintain viral suppression (achieved by most persons within 6 months of starting ART) can live long, healthy lives and pose effectively no risk of HIV transmission to their sexual partners.

**Methods:**

A model was used to estimate transmission rates in 2016 along the HIV continuum of care. Data for sexual and needle-sharing behaviors were obtained from National HIV Behavioral Surveillance. Estimated HIV prevalence, incidence, receipt of care, and viral suppression were obtained from National HIV Surveillance System data.

**Results:**

Overall, the HIV transmission rate was 3.5 per 100 person-years in 2016. Along the HIV continuum of care, the transmission rates from persons who were 1) acutely infected and unaware of their infection, 2) non-acutely infected and unaware, 3) aware of HIV infection but not in care, 4) receiving HIV care but not virally suppressed, and 5) taking ART and virally suppressed were 16.1, 8.4, 6.6, 6.1, and 0 per 100 person-years, respectively. The percentages of all transmissions generated by each group were 4.0%, 33.6%, 42.6%, 19.8%, and 0%, respectively.

**Conclusion:**

Approximately 80% of new HIV transmissions are from persons who do not know they have HIV infection or are not receiving regular care. Going forward, increasing the percentage of persons with HIV infection who have achieved viral suppression and do not transmit HIV will be critical for ending the HIV epidemic in the United States.

*On March 18, 2019, this report was posted as an *MMWR* Early Release on the *MMWR* website (https://www.cdc.gov/mmwr).*

## Introduction

Medical treatment has substantially improved the health, quality of life, and life expectancy of persons with HIV infection (*1*). The benefits of treatment are maximized with suppression of the virus (<200 copies of HIV/mL of blood on the most recent viral load test), which benefits health and decreases rates of transmission. Four recent studies found that viral suppression prevented sexual transmission of HIV (*2*–*5*). Together, these prospective studies found no HIV transmissions attributable to sex between HIV-discordant couples when the partner with HIV infection was on treatment and maintained viral suppression, despite documenting tens of thousands of acts of condomless sex in which the HIV-negative partner was not using preexposure prophylaxis. These findings indicate that HIV transmission can become a rare event if persons with infection can obtain treatment and achieve and maintain viral suppression. Today’s treatment regimens are simpler than those prescribed in the past, sometimes requiring only single-tablet formulations, with fewer side effects; most persons with HIV infection can achieve viral suppression within 6 months of initiating treatment. These findings also provide an important scientific underpinning to the new federal initiative headed by the U.S. Department of Health and Human Services (HHS) to end the HIV epidemic in the United States within 10 years (*6*).

Despite the availability of effective treatment, many of the 1.1 million persons with HIV infection in the United States are not effectively treated (*7*,*8*). In 2015, among all persons with HIV infection, 14.5% did not have a diagnosis, 37.2% were not in care,* and 48.9% were not virally suppressed (*7*). In addition, sexual and injection-drug–associated risk behaviors varied with knowledge of HIV infection status and access to care (*9*,*10*). Lack of effective treatment results in worse outcomes for persons with HIV infection and higher rates of HIV transmission and was associated with 38,700 new HIV infections in 2016 (*8*). To focus national and local prevention efforts to eliminate HIV, CDC used a model to estimate the number of persons and HIV transmissions at each step along the continuum of care.

## Methods

CDC updated the Progression and Transmission of HIV (PATH 2.0) model to estimate 2016 U.S. transmission rates by step along the HIV care continuum, population risk group, and age group (*9*). Mutually exclusive population risk groups included 1) men who have sex with men (MSM), 2) persons who inject drugs (men and women), 3) MSM who inject drugs, and 4) heterosexual men and women. PATH 2.0 tracked persons with HIV infection and their stage of disease (as measured by viral load and CD4 counts) as they moved along the HIV care continuum. Persons formed main and casual sexual partnerships as well as injection partnerships, with chances of transmission determined by sexual behaviors, injection risk behaviors, partnership preference, and viral load suppression status. Transmissions were tracked weekly in the acute stage (up to 3 months after HIV infection) and monthly thereafter. Persons newly infected with HIV were incorporated into the model.

Model inputs included behavioral data from National HIV Behavioral Surveillance and epidemiologic and clinical data from the National HIV Surveillance System. For persons in the model with viral suppression, the reduction in transmission rate was 100%^†^ (*2*–*5*). Based on population risk group and the cumulative amount of time spent in each age group and care-continuum step, the model estimated the number of infections each subgroup generated. The transmission rates (number of transmissions per 100 person-years) of a subgroup in a particular year were calculated by dividing the number of new infections generated by persons in that subgroup by the amount of time all persons spent in the subgroup and multiplying by 100. The trends in overall transmission rates estimated by the model were compared with the percentage of persons virally suppressed based on national HIV surveillance data.

## Results

**FIGURE 1:**
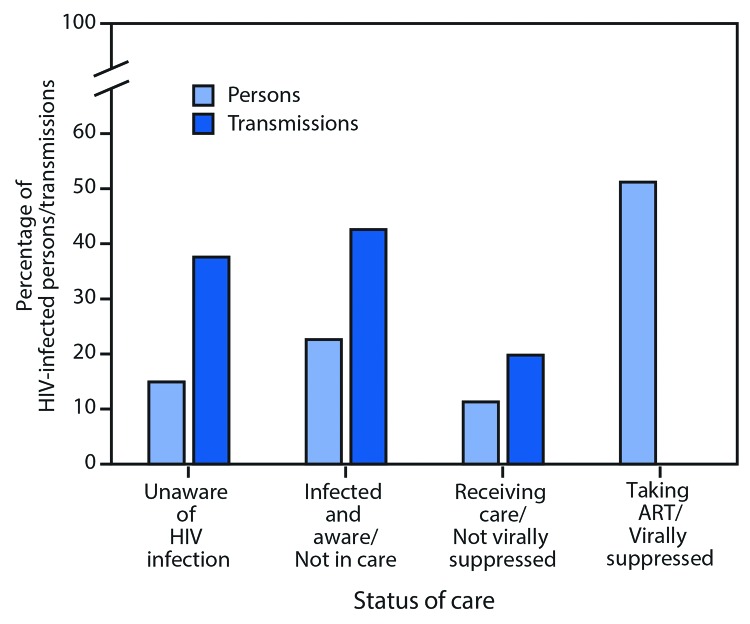
Percentage of persons* with human immunodeficiency virus (HIV) infection and transmissions along the continuum of HIV care^†^ — United States, 2016^§,¶^ **Abbreviation:** ART = antiretroviral therapy. * Percentage of persons in each subgroup averaged over 12 months in the model. ^†^ Receipt of medical care was defined as one or more tests (CD4 or viral load) in 2015. ^§^ Viral suppression was defined as <200 copies of HIV/mL of blood on the most recent viral load test. ^¶^ Unaware of HIV infection includes acutely infected and non-acutely infected persons unaware of their HIV infection.

The overall estimated HIV transmission rate in 2016 was 3.5 new infections per 100 person-years (Table). The rates of transmission decreased with progression along the HIV continuum of care. Persons who were acutely infected and unaware of their infection had the highest transmission rate (16.1), followed by persons who were non-acutely infected and unaware (8.4), those aware of their HIV infection but not in care (6.6), and those receiving HIV care but not virally suppressed (6.1). The rate was zero among those taking ART and virally suppressed. The percentage of all transmissions generated by each group was 4.0%, 33.6%, 42.6%, 19.8%, and 0%, respectively (Figure 1).

**TABLE:** Estimated number of persons with human immunodeficiency virus (HIV) infection and transmissions, by selected characteristics — United States, 2016

Characteristic	Transmission rate*	Persons in subgroup^†^ no. (%)	Transmissions generated^§^ no. (%)
**HIV care continuum**			
Unaware of HIV infection			
Acutely infected and unaware	16.1	9,600 (0.9)	1,500 (4.0)
Non-acutely infected and unaware	8.4	154,400 (14.0)	13,000 (33.6)
Aware of HIV infection			
Not in care	6.6	249,700 (22.6)	16,500 (42.6)
Receiving HIV care but not virally suppressed	6.1	125,300 (11.3)	7,700 (19.8)
Taking ART and virally suppressed	0.0	565,800 (51.2)	0 (0.0)
**Population risk group^¶^**			
MSM	4.4	645,600 (58.4)	28,300 (73.0)
Men who inject drugs	3.6	77,500 (7.1)	2,800 (7.2)
Women who inject drugs	2.2	46,600 (4.2)	1,000 (2.6)
MSM who inject drugs	3.8	53,400 (4.8)	2,100 (5.3)
Heterosexual men	2.7	87,500 (7.9)	2,400 (6.1)
Heterosexual women	1.2	194,200 (17.6)	2,300 (5.8)
**Age group (yrs)**			
13–24	5.1	65,200 (5.9)	3,300 (8.5)
25–34	4.6	160,900 (14.6)	7,300 (19.0)
35–44	3.9	214,800 (19.4)	8,400 (21.8)
45–54	3.2	258,500 (23.4)	8,200 (21.3)
≥55	2.8	405,500 (36.7)	11,400 (29.4)
**Total**	**3.5**	**1,104,900 (100)**	**38,700 (100)**

**Abbreviations:** ART = antiretroviral therapy; MSM = men who have sex with men.

* Number of transmissions per 100 person-years.

^†^ The number of persons in each subgroup in the model, averaged over 12 months and rounded to the nearest 100. Numbers might not sum to total because of rounding.

^§^ Generated from model and rounded to the nearest 100. Numbers might not sum to total because of rounding.

^¶^ MSM and persons who inject drugs can transmit sexually to men and women; persons who inject drugs can also transmit via injection drug use.

**FIGURE 2:**
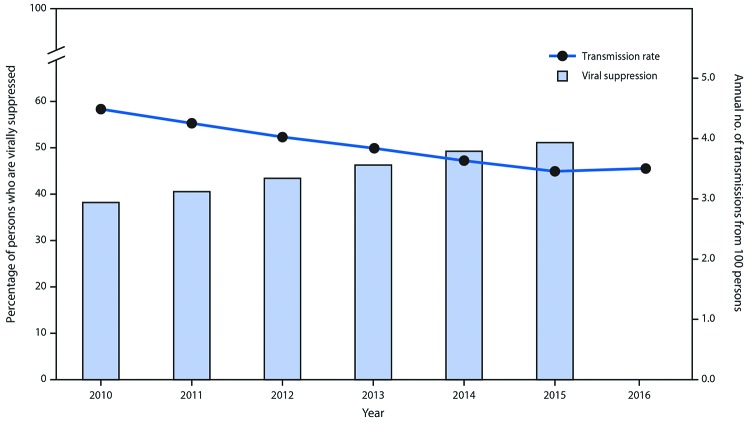
Percentage of persons with human immunodeficiency virus infection (HIV) who are virally suppressed* and HIV transmission rate^†^ — United States, 2010–2016^§^ * Viral suppression among persons with HIV infection; percentage obtained by multiplying the percentage with infection by the percentage virally suppressed among persons with diagnosed HIV. Viral suppression was defined as <200 copies of HIV/mL of blood on the most recent viral load test. ^†^ Generated from model. Measured in number of transmissions per 100 person-years (i.e., annual number of transmissions from 100 persons). ^§^ 2016 viral suppression data is not yet available.

Among estimated transmissions in 2016, 73.0% were from MSM, 9.7% from persons who inject drugs, 5.3% from MSM who inject drugs, and 12.0% from heterosexuals. The highest transmission rate (4.4 per 100 person-years) was among MSM, followed by MSM who inject drugs (3.8), men who inject drugs (3.6), heterosexual men (2.7), women who inject drugs (2.2), and heterosexual women (1.2). In general, the transmission rate was higher among younger persons and was highest among those aged 13–24 years (5.1). However, because of the size of the population, persons aged ≥55 years generated the largest percentage of new infections (29.4%). The model estimated a decline in overall transmission rate (from 4.5 to 3.5 per 100 person-years) from 2010 through 2016 that corresponded to a steady increase in viral suppression over those years (Figure 2).

## Discussion

The PATH 2.0 model estimated that HIV transmissions in 2016 occurred primarily from persons with HIV infection who did not know they were infected and persons with diagnosed HIV infection who were not in care; together, these two groups accounted for approximately 80% of new infections. Those who were in care but had not achieved viral suppression accounted for approximately 20% of transmissions. To end the HIV epidemic in the United States, the HHS initiative directs a path forward for success (*6*). First, early detection of HIV infection must be improved (*11*). Second, once HIV infection is identified, rapid entry into care and prevention services is crucial to ensure achievement of viral suppression as quickly as possible. Modeling studies indicate that viral suppression is critical for decreasing HIV incidence (*12*).

Providers play an important role in this effort by screening patients for HIV infection, actively linking and engaging persons with HIV infection into ongoing, comprehensive care, and emphasizing the importance of achieving and maintaining viral suppression for personal health and prevention benefits. Routine testing and targeted HIV testing are complementary approaches to addressing the 38% of transmissions that occurred from the estimated 15% of persons with undiagnosed HIV infection, by increasing awareness of HIV infection status and diagnosing infection sooner. Initial diagnosis is a necessary step to obtaining the benefits of HIV treatment and other psychosocial resources; however, the median interval between infection and diagnosis is 3 years (*11*). CDC recommends routine screening of all Americans aged 13–64 years at least once in their life and at least annual testing for those at high risk for acquiring HIV (*13*). Providers must work with their patients to ensure that HIV screening occurs in accordance with CDC guidelines. In addition, community partners can provide testing aimed at persons who are less likely to interact with the health care system on a regular basis. Together, these approaches can reduce undiagnosed HIV infection in the United States and thereby decrease transmission from persons with undiagnosed infection.

To address the 43% of transmissions that occur from the 23% of persons who have diagnosed infection and are not in care, improvements in rapid linkage to and retention in care are needed. Continued engagement in care might be difficult for some persons because of barriers that include lack of insurance, housing, transportation, or other resources; stigma and discrimination; mental health and substance use issues; and lack of trust in the medical system (*14*). These patients can benefit from tailored support services. Research on patterns of care over time could provide a better understanding of factors associated with patient dropout from care (*15*). Patients might respond well to knowledge of the personal and preventive benefits of treatment. Community efforts to increase public awareness of the benefits of viral suppression might help decrease stigma and make staying in care easier (*16*).

Helping patients adhere to treatment is important in addressing the 20% of infections that occur from the 11% of persons with HIV infection who are in care but not virally suppressed. Among persons with HIV infection who are in clinical care, approximately 80% were virally suppressed at their most recent visit (*17*,*18*), but about one third did not sustain viral suppression over a year (*17*,*18*). A tailored approach aimed at the barriers that are most relevant for the patient are important to improving adherence to medications and ultimately achieving and sustaining viral suppression.^§^

Among population risk groups within the model, most transmissions were from MSM because of the high proportion of persons with HIV infection who are MSM, the higher risk of transmission associated with anal sex, and high HIV infection prevalence among MSM. The highest transmission rate was among persons aged 13–24 years, and the highest number of transmissions were from persons aged ≥55 years, because of the larger number of persons living with HIV infection in this age group.

The findings in this report are subject to at least five limitations. First, PATH 2.0 required data on the sexual and injection behaviors of persons with HIV infection, and such data were limited (e.g., available data often were not stratified by age and disease stage) and mostly based on self-report. Second, although CDC assumed no injection drug use transmissions from persons who were virally suppressed, no data exist on the efficacy of viral suppression in reducing HIV transmission from injection drug use. Third, the model does not account for differences in prevalences of awareness of HIV infection and viral suppression by age. Thus, transmission rate estimates among younger persons might be underestimated, because data show a higher percentage of persons with HIV infection who were unaware of their infection (*8*) and a lower percentage with viral suppression among the younger age groups (*7*). Fourth, the model included 23,000 persons to represent the 1.1 million persons with HIV infection in the United States, and, for computational feasibility, the results obtained were scaled up to match current incidence. However, results were similar when a larger number of persons were input into the model. Finally, the model conservatively restricted reductions in transmission attributable to reduced viral load to those who achieved viral suppression. Some data indicate that, in general, persons with lower viral loads have a lower risk of transmission, even in the absence of viral suppression. However, data to determine viral loads over time for persons out of care or with undiagnosed HIV infection do not exist.

Although the prevalence of viral suppression among persons with HIV infection has been increasing, and the number of new infections and transmission rates have been decreasing in the United States, faster rates of change are needed to end the HIV epidemic in the United States. To accelerate progress, persons with HIV infection must receive a diagnosis soon after infection, begin treatment rapidly after diagnosis, adhere to treatment, and receive support services that help achieve and sustain viral suppression. Providers should screen patients for HIV infection at least once and test some patients more frequently; rapidly link, engage, or re-engage patients into comprehensive HIV care; and encourage patients to sustain viral suppression for their own health and because of the tremendous prevention benefits. In addition, many persons with HIV infection find it important to know that maintaining viral suppression prevents sexual transmission to partners, and sharing this knowledge more generally might decrease the stigma associated with HIV infection and help engage patients in consistent care.

**Summary**

**What is already known about this topic?**

Recent studies have demonstrated no human immunodeficiency virus (HIV) sexual transmission by persons whose infection is treated and who have achieved and sustain viral suppression. New estimates are needed to understand remaining sources of HIV transmissions.

**What is added by this report?**

An HIV transmission model indicated that, along the HIV care continuum, transmissions arise from persons with HIV infection who have not received a diagnosis or who have a diagnosed infection that is not controlled.

**What are the implications for public health practice?**

To control the spread of HIV in the United States, HIV infection must be diagnosed early and persons with HIV infection quickly engaged in sustained care and treatment.

## Footnotes

* Receipt of medical care is defined as one or more tests (CD4 or viral load) in the measurement year. The percentage of persons with HIV infection who are in care is obtained by multiplying the percentage with diagnosed infection by percentage in care among persons with diagnosed HIV infection.

† No data are available on the efficacy of viral suppression on reducing HIV transmission from injection drug use; 100% efficacy was assumed based on trial results for sexual transmission efficacy. Sensitivity analyses using an efficacy for injection-related transmission of 50%, 76%, and 90% resulted in transmission rates per 100 person-years of 0.13, 0.06, and 0.03, respectively.

§ https://www.cdc.gov/actagainstaids/campaigns/pic/index.html.
